# Development of a Head Acceleration Event Classification Algorithm for Female Rugby Union

**DOI:** 10.1007/s10439-023-03138-9

**Published:** 2023-02-09

**Authors:** David R. L. Powell, Freja J. Petrie, Paul D. Docherty, Hari Arora, Elisabeth M. P. Williams

**Affiliations:** 1 ZCCE, Faculty of Science and Engineering, Swansea University, Wales, UK; 2 Applied Sports, Technology, Exercise and Medicine Research Centre (A-STEM), Swansea University, Wales, UK; 3 Department of Mechanical Engineering, University of Canterbury, Christchurch, New Zealand; 4 Institute for Technical Medicine (ITeM), Furtwangen University, Villingen Schwenningen, Germany

**Keywords:** Machine learning, Head impact telemetry, Wearable sensors, Concussion, mTBI

## Abstract

Instrumented mouthguards have been used to detect head accelerations and record kinematic data in numerous sports. Each recording requires validation through time-consuming video verification. Classification algorithms have been posed to automatically categorise head acceleration events and spurious events. However, classification algorithms must be designed and/or validated for each combination of sport, sex and mouthguard system. This study provides the first algorithm to classify head acceleration data from exclusively female rugby union players. Mouthguards instrumented with kinematic sensors were given to 25 participants for six competitive rugby union matches in an inter-university league. Across all instrumented players, 214 impacts were recorded from 460 match-minutes. Matches were video recorded to enable retrospective labelling of genuine and spurious events. Four machine learning algorithms were trained on five matches to predict these labels, then tested on the sixth match. Of the four classifiers, the support vector machine achieved the best results, with area under the receiver operator curve (AUROC) and area under the precision recall curve (AUPRC) scores of 0.92 and 0.85 respectively, on the test data. These findings represent an important development for head impact telemetry in female sport, contributing to the safer participation and improving the reliability of head impact data collection within female contact sport.

## Introduction

Both concussive and sub-concussive head accelerations have been linked with acute and chronic neurocognitive changes.^23^ Due to the frequency of these head accelerations, sporting governing bodies have introduced law changes to reduce the number and severity of these events occurring in competition and training.^10,28,33^ For example, the international governing body, World Rugby, has implemented law changes to improve the safety of the tackle and to more harshly sanction those who fail to abide by the law.^28^ Given the complexity of concussion diagnosis and the subtlety of sub-concussive injuries, objective technologies can help to quantify the occurrence of potentially dangerous events.^19^

Head impact telemetry devices, a type of wearable kinematic sensors, have been implemented to provide objective measures of the frequency and severity of head acceleration events.^24^ These measurements can provide insights into the performance of interventions and help further our understanding of brain injury biomechanics to inform future interventions.^4,25^ Devices such headbands, patches, helmets, and Instrumented mouthguards have become commercially available for use in sports such as American football, football, and Australian rules football.^1,7,27,36^

Despite the burgeoning market for these devices, two significant issues persist with the utility of head impact telemetry devices for categorising head impacts. The first issue is the miss-estimation of head accelerations kinematics, and the second is the prevalence of false positive recordings.^15,17,27^ These issues negatively influence the devices capability to measure impact frequency and magnitude precisely and reliably; the two primary research interests.^17,26^ To mitigate this, video verification of impacts and machine learning algorithms have been proposed as methods to remove spurious events from datasets, although both have drawbacks.^27^

Video verification is a time-consuming activity with the quality of the output being dependant on the skill of the reviewers and the quality of the footage used.^8,27^ Furthermore, machine learning algorithms are reliant on high quality datasets, which in turn relies on both video verification and the quality data recorded by the head impact telemetry device. Additionally, questions remain over the on-field performance of algorithms trained on laboratory data.^17,27^ Despite this, it has been reported that only 36% of head impact studies use video verification, whilst 74% used filtering algorithms.^26^ Multiple studies have proposed machine learning algorithms to improve the on-field performance of instrumented mouthguards in specific sports, achieving near human-levels performance.^11,12,39,40^ This illustrates that for machine learning algorithms to become a viable injury detection method, it is imperative that they are derived from data collected from appropriate populations.

One area where the population differences may be particularly prevalent are between males and females, due to the differences in cervical spine and the kinematic response of the head during impact.^6,22,32,36^ The structure of the male cervical spine results in greater stability and resistance to external loading than the female cervical spine.^22^ This results in females experiencing an increased magnitude of displacement and acceleration during vehicle collisions and sporting contact events.^6,32,36^ As the motion of the head is reported to be different during different during these events, it is reasonable to assume that the trends developed by machine learning algorithms may be sex specific.

Despite the rapid growth of female rugby union participation and the increasing number of professional players, no algorithms for female sport or rugby union have been specifically developed.^37,38^ Therefore, it is imperative that this gap is addressed to ensure player safety and research quality.^36^ This study aims to develop a classification algorithm to detect head accelerations from instrumented mouthguards, in female rugby union players.

## Materials and Methods

### Data Collection

A rugby union head impact dataset was collected during the 2021/22 British Universities and Colleges Sports rugby season. The study was conducted within the framework outlined in the Consensus Head Acceleration Measurement Practices (CHAMP) 2022 project to ensure best practice.^3,29^ Instrumented mouthguards were issued to 25 players participating in six matches in the women’s premier south rugby division. All sensors remained functional during the six competitive matches, with data recorded from players representing all positions. Time-stamped videos were recorded from the centre line of the pitch with a 1080p, 30 fps device to enable the identification of events. Recordings were verified by two experienced reviewers, using a two-stage verification method. The times players were on-field were recorded, so that all off-field events could be excluded from the dataset. Two high certainty head acceleration events were found *via* video review and used to calibrate timing across the video and instrumented mouthguard data. A list of all events with corrected times was then compared to the video footage to verify the head acceleration events. For each event, if the player wearing the device that recorded the event was seen to be involved in a contact event, with a clear head acceleration within a ± 3 s window of the recorded time, the acceleration profiles would then be visually inspected for coherence. Visual inspection consisted of assessing the acceleration to determine whether it was representative of the on-video head acceleration, using criteria outlined within Williams *et al*.^36^ Events passing the verification process were labelled as positive events, whilst any on camera events that failed to pass were labelled as negative events. Institutional ethics approval was obtained prior to the commencement of the study, with subjects giving informed consent to their inclusion in the study (ethical approval number FP_01-10-21). All participants provided written informed consent prior to the start of the study, consistent with the approval granted by the Swansea University College of Engineering Research Ethics and Governance Committee.

Data were collected using the previously validated boil-and-bite instrumented mouthguards (Prevent Biometrics, Edina, MN, USA).^5,13,17,20^ All instrumented mouthguards contained a 3.2 kHz three-axis angular rate sensor, three 3.2 kHz single-axis linear accelerometers, a 130 mAh battery, proximity sensor, internal storage of up to 460 events and BLE transmission. The angular and linear sensors had measurement ranges between ± 35 rad s^−1^ and ± 200 *g*, respectively. When a head acceleration was detected, a segment of data from 10 ms prior, to + 40 ms post impact is stored temporarily on the instrumented mouthguard and transmitted *via* an iOS tablet on the side-line and uploaded to the Prevent Biometrics cloud server. Full descriptions of the technical specifications of the instrumented mouthguards have been reported in Ref. 5, 13, 17, 20.

The data transmitted to the Prevent cloud server is then fed to an algorithm to determine the cut-off frequency used to filter the recording. This is dependent on the strength of the noise recorded, which can be set to either 200, 100 or 50 Hz.^35^ As varying cut-off frequency filters would affect features used to train the classification algorithms, raw data was filtered in-house with a 200 Hz cut off frequency. The raw data included rotational velocity and linear acceleration and a proximity sensor reading. The proximity sensor data was used to provide a measure of the devices coupling with the teeth. Impacts that recorded low peak proximity values or large changes to the proximity reading were excluded from the study. Data containing low or inconsistent proximity values may have occurred off teeth or the device may have been subject to artefact during recording. Kinematic data was then filtered using a 200 Hz, 4th order Butterworth low-pass filter. Rotational acceleration was then estimated numerically from the rotational velocity, using four the neighbours of a central point to estimate the derivative, known as the five-point stencil derivative method.^31^ The absolute acceleration of each instance impact data was estimated *via* the Euclidean norm. Only events with a peak linear acceleration magnitude $\ge$ 9 *g* were used.

### Classifiers

Four classification algorithms were trained to determine whether the filtered accelerations were impact events or false impact events. Due to the large number of classification algorithms available, the classifiers selected for this task had previously shown success in head acceleration classification tasks. These algorithms were an adaptively boosted decision tree, support vector machine, and two extreme gradient boosted decision tree models, CatBoost and XGBoost, as used in Wu *et al*., Gabler *et al*., and Goodin *et al*.^11,12,39^

The classifier determined key patterns in the descriptive features of the filtered six-axis kinematic data. Features were grouped into four categories, pulse parameters, positional derivatives, power spectral density, and wavelet transformations. Except for positional derivatives, the feature categories have been used previously to train head acceleration event classifiers.^11,12,39^ Analysis was undertaken in a Python 3.8.10 computational framework.

### Pulse Parameters

The prominence, width, and number of pulses were identified from local maxima in the signal. The prominence of each peak was measured by calculating the vertical distance between the highest point and the lowest contour line. The width of each peak was measured by calculating the horizontal distance at the lowest contour line. In the event of there being multiple instances of either measure, the maximum value calculated would be used. The final measure was the total number of peaks per signal.

### Positional Derivatives

The first and second derivatives were calculated from each of the kinematic measurements. The first derivative was calculated from the change in two sequential recorded values, with the second derivative calculated by the same process from the first derivative. The maximum absolute value of each signal used, this provided the maximum rotational acceleration and jerk as calculated from the rotational velocity, and the respective jerk and snap as calculated from the linear and rotational accelerations.

### Power Spectral Density

The power spectral density describes the power of a signal in frequency components. This was calculated in 20 Hz windows using Welch’s method between 20 and 200 Hz, with the upper bound determined due to the filter’s cut-off frequency. The power values for each frequency were used as a feature, providing ten features per vector.

### Wavelet Transformation

A wavelet transformation was used to provide time dependant frequency analysis. A continuous wavelet transformation was conducted for each signal using the Ricker wavelet function between 10 and 200 Hz, in 10 Hz increments. The strength of frequencies calculated at the recording’s maxima were used as features.

The features were appended to a data frame, in which they were scaled to have zero mean and a standard deviation of one. To reduce overfitting and training times, the total number of features were reduced to 100 using the FCQ variant of the maximum relevance minimum redundancy method.^41^ There are no standardised rules on the appropriate number of features that should be considered. In this study, a value closer to the lower limit of features outlined Hua *et al*. 2005 was selected.^14^ This was to combat the prevalence of collinearity between the features of the dataset.^14^

### Performance Metrics

The area under the receiver operator curve (AUROC) and area under the precision recall curve (AUPRC) were used as the performance measures of the models. Area-based metrics measure the classifiers’ ability to discriminate between events at all decision thresholds, which in turn gives a measure of how well the classifier has separated the data.^21^ This provides greater detail of the classifier’s performance than measures that require a specific prediction threshold. To generate the curves, the true positive rate (recall), the precision and false positive rate were calculated at all decision thresholds. The true positive rate measures the classifier’s ability to correctly predict labels within the positive class (Eq. 1). Precision measures the quantity of true positive events from all predicted positive events (Eq. 2). Lastly, false positive rate is the measures the classifier’s ability to correctly label events from the negative class (Eq. 3).

AUROC uses true positive rate and false positive rate, which are calculated across decision threshold values between zero and one. These values are then plotted, with the resultant area under the curve is calculated. A score of 1.0 indicates the model will predict a correct label with 100% certainty every time, whereas 0.5 represents random performance. AUPRC similarly calculates the area under a curve, however, the plot displays the true positive rate (recall) against precision. It has previously been identified as an important metric in head impact telemetry due to its value when evaluating imbalanced datasets.^30,39,40^ The area under the curve is equal to the average of the precision of the classifier across all decision thresholds. Should a model achieve perfect classification results, then an area under the curve score of 1.0 would be returned. Conversely, a classifier with no classification skill would return a score equal to the fraction of positive events within the dataset.

1 $$Recall, \,True\,Positive\,Rate = \frac{TP}{{TP + FN}}$$

2 $$Precision = \frac{TP}{{TP + FP}}$$

3 $$False\,Positive \,Rate = \frac{FP}{{FP + TN}}$$

Shapley additive explanation (SHAP) values were calculated to analyse the effect of feature values on the classifier’s outputs.^18^ SHAP values are calculated from the prediction of labels on feature vectors constructed from feature values and combinations found within the dataset. The effect of the feature value can then be measured along with its effect on label prediction.

### Classifier Selection and Development

The rugby union data was roughly split into a training group using five of the six matches (~ 80%), and data from one game was used as the testing group. No data from the same session appeared in both the training and test groups.

The first training stage was the simultaneous hyper-parameter and feature selection, conducted using an eightfold cross-validation. During cross-validation, the training data was split into eight approximately equal groups, with all but one of the groups used to train the classifier and the remaining groups used to assess the classifier’s performance. The cross-validation process works so that all data appears in the validation group once. A dictionary of hyper-parameters was created, and an exhaustive search of all combinations was used to find the highest performing classifier hyper-parameters, with models optimised for AUROC. This was initially tested with ten features then re-run with ten features added, until the feature set had reached the maximum size of 100 features. Feature optimisation was conducted in this manner to reduce the likelihood of overtraining the classifiers, whilst optimising the features and hyper-parameters. The hyper-parameters of the highest performing models were then recorded. These configurations were then retrained on the entire training dataset ten times, with the highest performing model then used to predict labels on the test data. AUROC, AUPRC and SHAP values were then calculated to assess and explain model performance. The code and trained classifiers may be made available following a reasonable request to the corresponding author.

## Results

A total of 214 head acceleration events and 466 spurious events were identified from the raw data during the video verification process. The training data was formed of five matches, containing 166 head accelerations events and 400 spurious events, with the remainders used for the test data.

The 100 features selected using the maximum relevance minimum redundancy method included features from each category and kinematic measurement, over a wide range of frequencies. This consisted of 20 pulse parameters predominantly measuring the rotational acceleration and velocity, nine higher frequency power spectral density measures of linear acceleration, and eight derivatives of rotational acceleration and velocity. The remaining features consist of wavelet transformation from all kinematic measurements across the whole spectrum of frequencies used. A summary of the most informative 25 features obtained is shown in Table 1.

**Table 1:** Highest performing features and their orientation on the head impact classifier models.

Feature category	Recording	Direction and frequency			
		*X*	*Y*	*Z*	*R*
Wavelet transformations	Linear acceleration	200, 160	20, 30	10, 50, 30, 40	
	Rotational velocity	100, 50, 60, 80	160		100, 80
	Rotational acceleration		30		
Positional derivatives	Rotational velocity	PP, PN, PW	PW	PN, PW	PW, PN
	Rotational acceleration	PP	PW		

**Figure 1:**
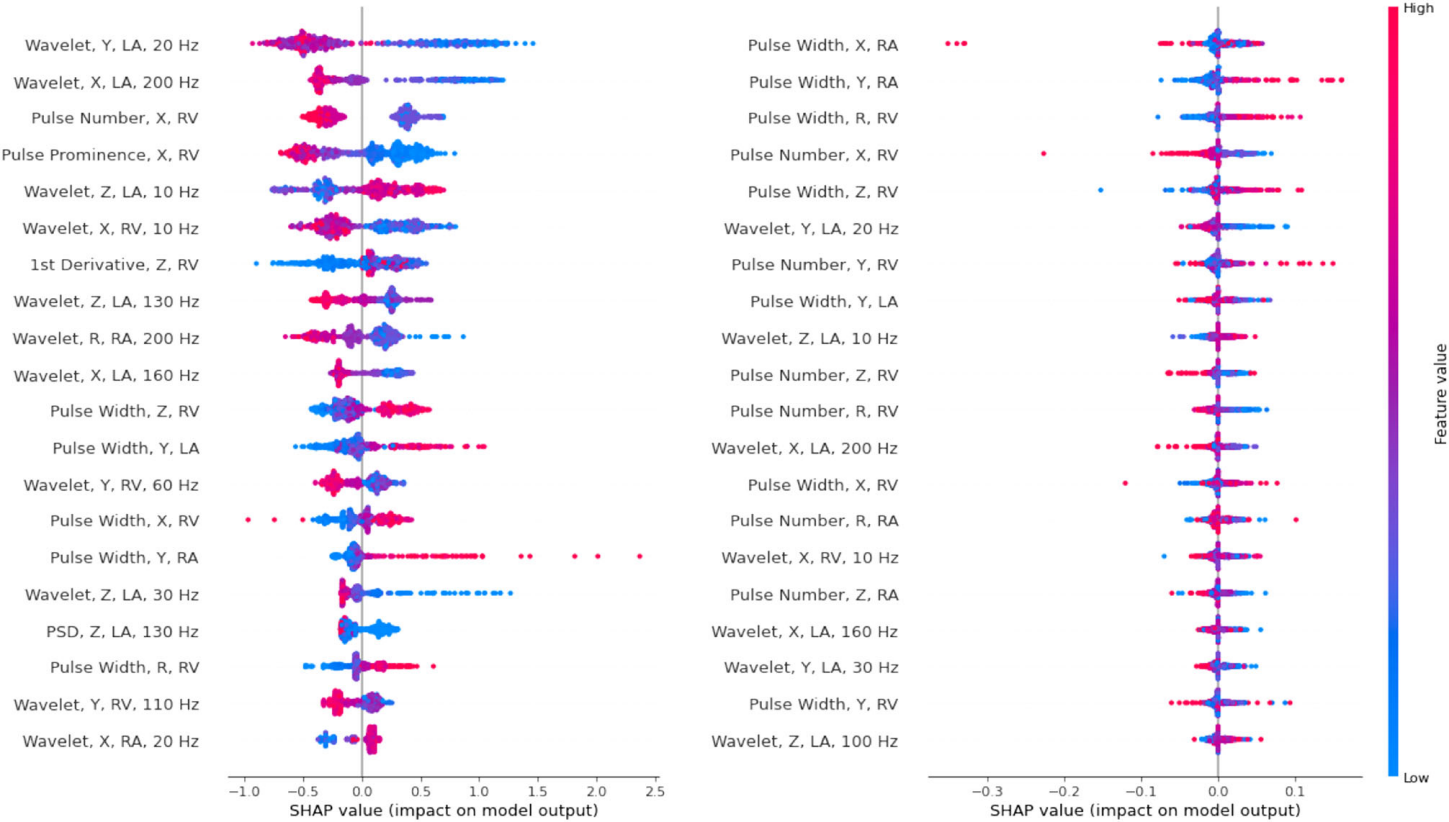
The 20 most valuable features identified through SHAP for classification. (Left = CatBoost classifier, Right = Support vector machine).

The best performing 20 features for the CatBoost and SVM classifiers as identified by SHAP values were found. The most important features for classification were predominantly pulse parameters and wavelet transformations at very low or high frequencies. For the CatBoost classifier, wavelet transformations made up over half of the 20 features used. The transformations in the *X* direction at 20 Hz and *Y* direction at 200 Hz for the linear classifier were the most important features. These important features and their relative contributions to classification of head impacts are provided in Fig. 1. SHAP values were also calculated for the SVM classifier, with the top 20 features primarily consisted of pulse parameters, with the remainder of features being wavelet transformations, the results are shown in Fig. 1.

**Figure 2:**
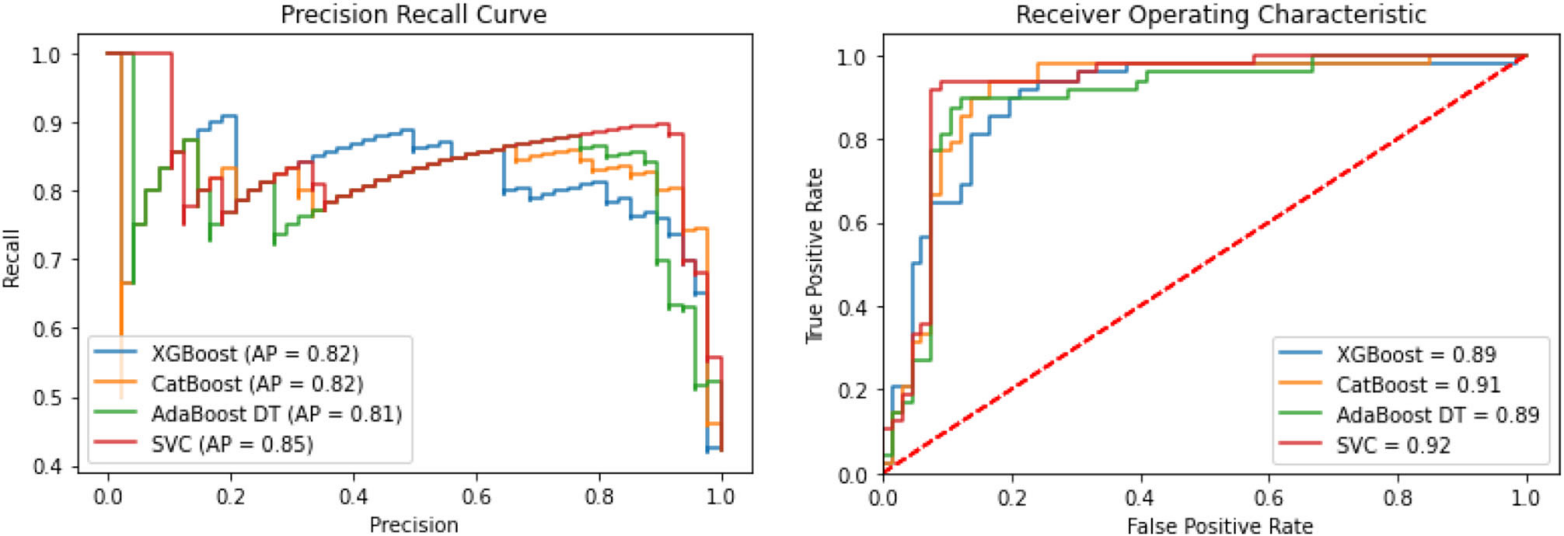
Left, classifier precision recall curves. Right, classifier receiver operator curves.

During the optimisation process, the CatBoost classifier achieved the highest cross-validation AUROC score of 0.900, with the next highest performing classifiers being XGBoost, SVM and Adaboost decision tree respectively. When tested upon the test dataset, the SVM classifier achieved the highest AUROC and AUPRC, followed by the CatBoost, XGBoost and Adaboost DT. The results of all the tests are shown in Table 2, with all receiver operator curves and precision recall curves plotted in Fig. 2.

**Table 2:** Classification performance of various models in cross-validation and validation within the test dataset.

Model	Cross-validation optimisation Score (AUROC)	Test score AUROC	Test score AUPRC
SVM (70 features)	0.885	0.92	0.85
Adaboost DT (100 features)	0.866	0.89	0.81
XGBoost (20 features)	0.892	0.89	0.82
CatBoost (90 features)	0.900	0.91	0.82

## Discussion

In this study the performance of four classification algorithms trained on head impact telemetry data from women’s collegiate rugby union were evaluated. This work developed a classification algorithm to detect head accelerations recorded by instrumented mouthguards, in female rugby union players. In total, four head acceleration classification models were developed. This is the first study to both develop classifiers with exclusively female data, and to classify impacts from rugby union. Each model performed well in the classification task, with a support vector machine algorithm providing the greatest performance when tested, with an AUROC and AUPRC of 0.92 and 0.85 respectively. This study represents an important step in the development of female specific head acceleration detection algorithms, which will contribute to safer participation in rugby union and more reliable study of female contact sport.

SHAP and mRMR feature analysis identified pulse parameters and wavelet transformations as the most valuable feature categories, providing both the greatest number, and most powerful features for classification (Fig. 1, Table 1). The frequencies used to train the classifiers ranged from 10 to 200 Hz, with features closer to the 10 and 200 Hz limits typically appearing earlier in the order of selection. Both high and low frequencies contributed to predictions of the negative classes (Table 1). High feature values of wavelet transformations that had characteristic frequencies at the feature limits generally led to negative class label prediction. Conversely, features that led to positive prediction were limited to those based on low frequencies and some pulse parameters. For example, 10 Hz motion in the vertical direction (*z*) was predictive of actual events, as was pulse width in all directions (Fig. 1).

This analysis highlighted the importance of the feature’s direction and the characteristic frequency. A feature with characteristic frequency of 20 Hz in the y-direction, for example, was strongly negative in its contribution to prediction of actual impacts, whereas a feature with characteristic frequency of 10 Hz in the *z* direction was strongly positive in its prediction of actual impact (Fig. 1). This may occur as the low frequency features may be capturing voluntary human motion, which generally occurs at frequencies below 10 Hz.^16^ For example, spurious events can occur while players are at rest and motion largely restricted to the *x* and *y* axis. As a player runs or enters contact, there will be a greater movement in the *z* direction, which is registered more often in positive events, hence leading to a positive label prediction.

This study used a novel data set collected from 25 iMGs from adult female rugby players in a university, 1st team squad. This data was collected in accordance with the methodology outlined by Williams *et al*..^36^ The last six games of the season were monitored, with the number of players in the match day squad possessing iMGs ranging from 12 to 22 per game. In total, over 7500 player minutes of data was gathered, with every field position represented. Hence, the data is broadly representative of female rugby at the penultimate level. Each game was verified independently by two reviewers, who while blinded to each-other’s classifications, achieved a high level of agreement. In total 680 impacts were used within the training and testing of the classification algorithms, which is in line with the datasets used in the studies of Wu *et al*. and Gabler *et al*. ^11,39^ The Prevent boil-and-bite instrumented mouthguards performed comparably to the on-field performance tests reported in Kieffer *et al*. ^17^ The algorithms saw consistent performance across the training and test datasets reaching values proximal with those in the literature, indicating a reliable study.

The classification results shown in Table 2 are, by some metrics, slightly lower than some studies of American football and Australian rules football head accelerations.^9,11,12,39^ Specifically, Goodin *et al*. recorded maximum recall of 94.7 and 95.7% for genuine and spurious events in Australian rules football.^12^ Whilst the American football classifiers of, Wu *et al*., Gabler *et al*., and Domel *et al*. achieved precisions of 98.3, 93.8 and 86.0, with recall values of 87.2, 100 and 76% respectively.^9,11,39^ For comparison, In this study a peak precision of 89.9% and recall of 91.1% were achieved with the SVM classifier. Note that the comparisons across sports lack equipoise to make summative assessments of the algorithms considered. In particular, classification of head accelerations in rugby union requires capture of a variety of head acceleration types. Hence, such models need to be broad, with many features to capture the various head acceleration types. This model broadness provides more potential for spurious data to correlate with one of the features, and lead to a false positive. It is reasonable to assume that sports with a limited range of head acceleration types can classify events with fewer features and limit the potential for false correlation. The complexity of head accelerations in rugby union may also manifest as classification errors during the video verification process, as matches were typically filmed without redundancy and from the side-line. While some events were excluded due to being unclear in the match footage, some events may have been misconstrued as the incorrect label by the video verifiers. Additionally, some phases of play such as tackling, rucking, and mauling occur can lead to multiple recordings during the event, which in turn makes classification difficult. In these events, the recording where no clear movement of the head could be seen on camera were excluded from the study. This resulted in a dataset containing only events recorded when clear head acceleration was seen, and events with no head acceleration. Some previous studies have included only direct head contacts, so perhaps the inclusion of indirect events led to the inclusion of more spurious events or a more difficult classification task.^11,39^ A further limitation to this study, is the lack of availability of testing data acquired from a separate cohort. Whilst the results were consistent across the cross validation and testing, further validation from new end users would assist in assuring the generalisability of the developed algorithms to female rugby union cohorts. Furthermore, care should be taken when extrapolating the results of this analysis beyond the cohort tested. In particular, these results may not be applicable in adolescent female rugby, in the professional levels, or in other women’s sports. With the classifiers previously validated in other sports, it appears that decision tree-based ensemble and support vector machine algorithms are both capable of creating high performing classifiers.^11,12,20^ The maximum relevance minimum redundancy method was used to reduce the feature count from 540 to 100 to aid classifier training. This proved successful as the features identified as most important through mRMR were later confirmed as highly valuable by the SHAPley additive explanations (Fig. 1). Figure 2 shows the SHAP values of the most valuable feature in the Catboost classifier (Wavelet Transform, Y, Lin Acc, 20 Hz) was far more powerful than the 20th most predictive feature. Hence, the pruning approach that was utilized to mitigate overfitting, was unlikely to lose significant amounts valuable information. This retention of performance is shown in Fig. 2. Whilst this unbalanced contribution of feature strength wasn’t as pronounced in the support vector machine approach, there was still a reduction in feature power across the top 20. Overall, the distinct SHAP values for each feature across the four algorithms tested highlights the importance of providing the algorithms with diverse array of features for head impact classification tasks.

This study has illustrated that it is possible to create high performing head acceleration event classifiers for female rugby union. This will aid future researchers to more quickly and accurately identity head acceleration events within female rugby union. It has been previously reported that there are sex differences between the male and female cervical spine and sex differences in measured head peak-kinematics during rugby union matches.^2,22,34,36^ Further to this, there has been no cross-validation study of head acceleration classification algorithms across sports or sexes. Such cross-validation is required to establish the repeatability and generalisability of model-based interpretation of instrumented mouthguard data for head acceleration monitoring. Until this is done, it is reasonable to assume that there may also be differences in impact characteristics and that a “one size fits all” approach to classification is not appropriate. As head acceleration classification algorithms learn specific patterns and trends, the patterns learnt from male sport, may not provide replicable results in female sport. Failing to address this may lead to significant differences in the understanding and identification of brain injury in female sport. With the rapid growth and progressive professionalisation of female rugby union, it is essential to create specific state-of-the-art head acceleration classification models to provide reliable data, and to protect players.^37,38^

## References

[1] Allison MA, Kang YS, Bolte JH, Maltese MR, Arbogast KB (2014). Validation of a Helmet-based system to measure head impact biomechanics in ice hockey. Med. Sci. Sports Exerc..

[2] Alsalaheen B, Landel R, Hunter-Giordano A, Shimamura KK, Quatman-Yates C, Hanke T, McCulloch KL (2019). A treatment-based profiling model for physical therapy management of patients following a concussive event. J. Orthop. Sports Phys. Therapy.

[3] Arbogast KB, Caccese JB, Buckley TA, McIntosh AS, Henderson K, Stemper BD, Solomon G, Broglio SP, Funk JR, Crandall JR (2022). Consensus head acceleration measurement practices (CHAMP): origins, methods transparency and disclosure. Ann. Biomed. Eng..

[4] Asken BM, Brooke ZS, Stevens TC, Silvestri PG, Graham MJ, Jaffee MS, Clugston JR (2019). Drill-specific head impacts in collegiate football practice: implications for reducing “friendly fire” exposure. Ann. Biomed. Eng..

[5] Bartsch, A. J., S. Samorezov, E. Benzel, V. Miele, and D. Brett. Validation of an “intelligent Mouthguard” Single Event Head Impact Dosimeter. *SAE Technical Papers* 2014-Novem, 2014. 10.4271/2014-22-0001 26192948

[6] Caccese JB, Buckley TA, Tierney RT, Rose WC, Glutting JJ, Kaminski TW (2018). Sex and age differences in head acceleration during purposeful soccer heading. Res. Sports Med..

[7] Carey L, Stanwell P, Terry DP, McIntosh AS, Caswell SV, Iverson GL, Gardner AJ (2019). Verifying head impacts recorded by a wearable sensor using video footage in rugby league: a preliminary study. Sports Med Open.

[8] Cortes N, Lincoln AE, Myer GD, Hepburn L, Higgins M, Putukian M, Caswell SV (2017). Video analysis verification of head impact events measured by wearable sensors. Am. J. Sports Med..

[9] Domel AG, Raymond SJ, Giordano C, Liu Y, Yousefsani SA, Fanton M, Cecchi NJ, Vovk O, Pirozzi I, Kight A, Avery B, Boumis A, Fetters T, Jandu S, Mehring WM, Monga S, Mouchawar N, Rangel I, Rice E, Roy P, Sami S, Singh H, Wu L, Kuo C, Zeineh M, Grant G, Camarillo DB (2021). A new open-access platform for measuring and sharing mTBI data. Sci. Rep..

[10] Emery CA, Black AM, Kolstad A, Martinez G, Nettel-Aguirre A, Engebretsen L, Johnston K, Kissick J, Maddocks D, Tator C, Aubry M, Dvořák J, Nagahiro S, Schneider K (2017). What strategies can be used to effectively reduce the risk of concussion in sport? A systematic review. Br. J. Sports Med..

[11] Gabler L, Huddlestone S, Dau N, Lessley D, Arbogast KB, Thompson X, Resch J, Crandall J (2020). On-field performance of an instrumented mouthguard for detecting head impacts in American Football. Ann. Biomed. Eng..

[12] Goodin P, Gardner AJ, Dokani N, Nizette B, Ahmadizadeh S, Edwards S, Iverson GL (2021). Development of a machine-learning-based classifier for the identification of head and body impacts in elite level Australian rules football players. Front. Sports Act Living.

[13] Hedin, D. S., P. L. Gibson, A. J. Bartsch, and S. Samorezov. Development of a head impact monitoring “Intelligent Mouthguard.” 2016. 10.1109/EMBC.2016.7591119. 10.1109/EMBC.2016.7591119 28268724

[14] Hua J, Xiong Z, Lowey J, Suh E, Dougherty ER (2005). Optimal number of features as a function of sample size for various classification rules. Bioinformatics.

[15] Jadischke R, Viano DC, Dau N, King AI, McCarthy J (2013). On the accuracy of the head impact telemetry (HIT) system used in football helmets. J Biomech.

[16] Khusainov R, Azzi D, Achumba I, Bersch S (2013). Real-time human ambulation, activity, and physiological monitoring: taxonomy of issues, techniques, applications. Chall. Limit. Sens..

[17] Kieffer EE, Begonia MT, Tyson AM, Rowson S (2020). A two-phased approach to quantifying head impact sensor accuracy: in-laboratory and on-field assessments. Ann. Biomed. Eng..

[18] Lundberg, S. M., P. G. Allen, and S.-I. Lee. A Unified Approach to Interpreting Model Predictionsat. https://github.com/slundberg/shap

[19] McCrory PR (2017). Consensus statement on concussion in sport—the 5th international conference on concussion in sport held in Berlin, October 2016. Br. J. Sports Med..

[20] Miller LE, Kuo C, Wu LC, Urban JE, Camarillo DB, Stitzel JD (2018). Validation of a custom instrumented retainer form factor for measuring linear and angular head impact kinematics. J. Biomech. Eng..

[21] Mitchell TM (1997). Machine Learning.

[22] Mohan M, Huynh L (2019). Sex differences in the spine. Curr. Phys. Med. Rehabil. Rep..

[23] Nowinski CJ, Bureau SC, Buckland ME, Curtis MA, Daneshvar DH, Faull RLM, Grinberg LT, Hill-Yardin EL, Murray HC, Pearce AJ, Suter CM, White AJ, Finkel AM, Cantu RC (2022). Applying the Bradford Hill criteria for causation to repetitive head impacts and chronic traumatic encephalopathy. Front. Neurol..

[24] O’Connor KL, Rowson S, Duma SM, Broglio SP (2017). Head-impact-measurement devices: a systematic review. J. Athl. Train..

[25] Patton DA (2016). A review of instrumented equipment to investigate head impacts in sport. Appl. Bionics. Biomech..

[26] Patton DA, Huber CM, Jain D, Myers RK, McDonald CC, Margulies SS, Master CL, Arbogast KB (2020). Head impact sensor studies in sports: a systematic review of exposure confirmation methods. Ann. Biomed. Eng..

[27] Patton DA, Huber CM, McDonald CC, Margulies SS, Master CL, Arbogast KB (2020). Video confirmation of head impact sensor data from high school soccer players. Am. J. Sports Med..

[28] Raftery M, Tucker R, Falvey ÉC (2021). Getting tough on concussion: how welfare-driven law change may improve player safety—a Rugby Union experience. Br. J. Sports Med..

[29] Rowson S, Mihalik J, Urban J, Schmidt J, Marshall S, Harezlak J, Stemper BD, McCrea M, Funk J (2022). Consensus head acceleration measurement practices (CHAMP): study design and statistical analysis. Ann. Biomed. Eng..

[30] Saito T, Rehmsmeier M (2015). The precision-recall plot is more informative than the ROC plot when evaluating binary classifiers on imbalanced datasets. PLoS ONE.

[31] Sauer T (2012). Numerical Analysis.

[32] Stemper BD, Derosia JJ, Yogananan N, Pintar FA, Shender BS, Paskoff GR (2009). Gender dependent cervical spine anatomical differences in size-matched volunteers-biomed 2009. Biomed. Sci. Instrum..

[33] Stemper BD, Shah AS, Harezlak J, Rowson S, Duma S, Mihalik JP, Riggen LD, Brooks A, Cameron KL, Giza CC, Houston MN, Jackson J, Posner MA, McGinty G, DiFiori J, Broglio SP, McAllister TW, McCrea M (2019). Repetitive head impact exposure in College Football following an NCAA rule change to eliminate two-a-day preseason practices: a study from the NCAA-DoD CARE Consortium. Ann. Biomed. Eng..

[34] Stemper BD, Yoganandan N, Gennarelli TA, Pintar FA (2005). Localized cervical facet joint kinematics under physiological and whiplash loading. J. Neurosurg. Spine.

[35] Tooby J, Weaving D, Al-Dawoud M, Tierney G (2022). Quantification of head acceleration events in rugby league: an instrumented mouthguard and video analysis pilot study. Sensors.

[36] Williams EMP, Petrie FJ, Pennington TN, Powell DRL, Arora H, Mackintosh KA, Greybe DG (2021). Sex differences in neck strength and head impact kinematics in university rugby union players. Eur. J. Sport Sci..

[37] Woodhouse LN, Tallent J, Patterson SD, Waldron M (2022). International female rugby union players’ anthropometric and physical performance characteristics: a five-year longitudinal analysis by individual positional groups. J. Sports Sci..

[38] World Rugby. World Rugby Year in Review 2018. 2019.

[39] Wu LC, Kuo C, Loza J, Kurt M, Laksari K, Yanez LZ, Senif D, Anderson SC, Miller LE, Urban JE, Stitzel JD, Camarillo DB (2018). Detection of American Football head impacts using biomechanical features and support vector machine classification. Sci. Rep..

[40] Wu LC, Zarnescu L, Nangia V, Cam B, Camarillo DB (2014). A head impact detection system using SVM classification and proximity sensing in an instrumented mouthguard. IEEE Trans. Biomed. Eng..

[41] Zhao, Z., R. Anand, and M. Wang. Maximum relevance and minimum redundancy feature selection methods for a marketing machine learning platform. 2019. 10.1109/DSAA.2019.00059.

